# Dark citations to Federal resources and their contribution to the public health literature

**DOI:** 10.3389/frma.2023.1235208

**Published:** 2023-08-29

**Authors:** Jessica M. Keralis, Juan Albertorio-Díaz, Travis Hoppe

**Affiliations:** National Center for Health Statistics, U.S. Centers for Disease Control and Prevention, Hyattsville, MD, United States

**Keywords:** dark citations, bibliometrics, non-indexed information resources, web-based resources, governmental resources

## Abstract

The term “dark citations,” which has been previously used to refer to citations of information products outside of traditional peer-reviewed journal articles, is adapted here to refer to those that are not linked to a known indexed identifier and are effectively invisible to traditional bibliometric analysis. We investigate an unexplored source of citations in the biomedical and public health literature by surveying the extent of dark citations across the U.S. government. We systematically focus on public health, quantify their occurrences across the government, and provide a comprehensive dataset for all dark citations within PubMed.

## 1. Introduction

Bibliometrics, or quantitative evaluation based on citations is widely used by research institutions to evaluate research performance and assess impact.^1^ By constructing a metric related directly to citations (Jensen, 2016), this type of evaluation can be used by funding organizations to estimate the influence and knowledge contribution of a grantee to the academic literature. Common identifiers include the International Standard Book Number (ISBN) for books (International ISBN Agency, 2017), the International Standard Serial Number (ISSN) for magazines and other serial publications (ISSN InterNational Centre, 2015), U.S. Patent and Trademark Office (USPTO) number for patents, clinical trials, digital object identifiers (DOI) (International DOI Foundation., 2019) for peer-reviewed journal articles and other selected publications.

The U.S. federal government funds over $137 billion dollars toward basic, applied research and development (Congressional Research Service, 2022). While the direct output of this research is often cataloged and tracked through an end product like a publication, patent, book, or clinical trial, not all products can be readily tracked with an identifier. Federal agencies often release authoritative information through guidelines, fact sheets, manuals, web pages, and other informational products that are not systematically indexed, referred to as “dark citations” (Jensen, 2016). Tracking the reach and usage of these resources is critical to assess impact.

This works aims to systematically survey the extent and quantify dark citations across the U.S. government with a focus on public health. In this work, we operationally define the term “dark citation” as any citation that does not include an indexed representation. To focus on federally funded resources, we restricted our analysis to citations whose reference text included a uniform resource locator (URL) pointing to the top-level U.S. governmental domain (.gov).

## 2. Methods

We downloaded the entirety of the PubMed database^2^ on June 6, 2022, in XML format, including both the baseline and update files. We merged the records keeping the most updated information for each PubMed IDentifer (PMID) resulting in 35,408,546 records. We filtered PMIDs that lacked a <referencelist> element tag, as these publications lacked reference information leaving 9,223,992 publications. For each reference, we eliminated those with element tags that included a PMID, PMCID, DOI, or PII element as these directly linked to an indexed article. The remaining references were parsed as free text and we scanned for URLs. For a reference to be considered a dark citation in this work it must include a URL with a top-level .gov domain, and not reference PubMed itself, clinicaltrials.gov, or paft.uspto.gov. For NIH, we folded domains that belonged to the agency, like cancer.gov. Illustrative examples are provided in the Supplementary material. We generated descriptive statistics displaying the number and percentage of dark citations among U.S. Department of Health and Human Services (HHS) agencies,^3^ because HHS comprised the largest share of dark citations among federal executive agencies (Table 1); the number and percentage of dark citations among U.S. Centers for Disease Control and Prevention (CDC) centers, because CDC comprised the largest share of dark citations among HHS sub-agencies (Table 2); and number and percentage of dark citations among National Center for Health Statistics (NCHS) divisions, because NCHS comprised the largest share of dark citations among CDC centers (Table 3).

**Table 1:** Number and percentage of dark citations in PubMed from the Federal executive branch, by Department.

**Federal executive agency**	**Acronym**	**Dark citations**	**Percent**
Department of Health and Human Services	HHS	71,664	74.3%
Department of Commerce		5,329	5.5%
Environmental Protection Agency	EPA	3,602	3.7%
United States Department of Agriculture	USDA	2,618	2.7%
Department of Labor	DOL	1,932	2.0%
Veteran Affairs	VA	1,694	1.8%
Department of Justice	DOJ	1,340	1.4%
Department of Education		1,209	1.3%
Department of Interior		1,091	1.1%
Department of Energy		902	0.9%
National Archives and Records Administration	NARA	805	0.8%
National Aeronautics and Space Administration	NASA	602	0.6%
Department of Homeland Security	DHS	369	0.4%
United States Agency for International Development	USAID	333	0.3%
Department of Transportation	DOT	317	0.3%
General Service Administration	GSA	291	0.3%
Educational Opportunity Program	EOP	287	0.3%
Interagency		275	0.3%
Department of Housing and Urban Development	HUD	99	0.1%
Department of Treasury	Treasury	88	0.1%
Department of Defense	DOD	62	0.1%
Other	Other	1,258	1.3%

**Table 2:** Number and percentage of dark citations in PubMed from the U.S. Department of Health and Human Services (HHS), by sub-agency.

**HHS agency**	**Acronym**	**Dark citations**	**Percent**
Centers for Disease Control and Prevention	CDC	25,314	35.32%
Food and Drug Administration	FDA	18,461	25.76%
National Institutes of Health	NIH	11,404	15.91%
Center for Medicare and Medicaid Services	CMS	4,515	6.30%
HHS Office of the Secretary	OS	4,082	5.70%
Agency for Healthcare Research and Quality	AHRQ	3,723	5.20%
Health Resources and Services Administration	HRSA	1,944	2.71%
Substance Abuse and Mental Health Services Administration	SAMHSA	1,918	2.68%
Administration of Community Living	ACL	99	0.14%
Indian Health Service	IHS	73	0.10%
Administration for Strategic Preparedness and Response	ASPR	71	0.10%
Administration for Children and Families	ACF	60	0.08%

**Table 3:** Number and percent of dark citations in PubMed from the CDC, by center, office, institute, system, or publication set.

**CDC center, institute, office, system, or publication set**	**Acronym**	**Dark citations**	**Percent**
Coronavirus Disease	COVID	4,147	16.58%
National Center for Health Statistics	NCHS	3,907	15.62%
National Center for Chronic Disease Prevention and Health Promotion	NCCDPHP	3,698	14.78%
National Center for HIV, Viral Hepatitis, STD, and Tuberculosis Prevention	NCHHSTP	2,700	10.79%
National Center for Emerging and Zoonotic Infectious Diseases	NCEZID	2,206	8.82%
National Center for Injury Prevention and Control	NCIPC	1,843	7.37%
National Center for Immunization and Respiratory Diseases	NCIRD	1,338	5.35%
Morbidity and Mortality Weekly Report	MMWR	886	3.54%
Agency for Toxic Substances and Disease Registry	ATSDR	680	2.72%
National Institute for Occupational Safety and Health	NIOSH	639	2.55%
National Center on Birth Defects and Developmental Disabilities	NCBDDD	451	1.80%
Office of the Director	OD	438	1.75%
Wide-ranging ONline Data for Epidemiologic Research	WONDER	351	1.40%
National Center for Environmental Health	NCEH	281	1.12%
CDC Library		270	1.08%
Center for Preparedness and Response	CPR	259	1.04%
Center for Surveillance, Epidemiology, and Laboratory Services	CSELS	243	0.97%
Center for Global Health	CGH	242	0.97%
Office of Minority Health and Health Equity	OMHHE	101	0.40%
File Transfer Protocol	FTP	93	0.37%
Office of the Secretary	OS	75	0.30%
Center for State, Tribal, Local, and Territorial Support	CSTLTS	69	0.28%
Socrata (data.cdc.gov backend)		46	0.18%
Severe acute respiratory syndrome	SARS	20	0.08%
Deputy Director Public Health Science and Surveillance	DDPHSS	17	0.07%
Office of Laboratory Science and Safety	OLSS	11	0.04%
Occupational and Public Health Specialty Section	OPHSS	8	0.03%

For each .gov URL collected from a dark citation, we determined provenance (e.g., branch, department, State, agency, etc.) by matching the domain against the registrar of U.S. government domains^4^ provided by the Cybersecurity and Infrastructure Security Agency. To identify the status of the links, we programmatically accessed each link a total of 5 times over the course of a month. A link was considered valid if it returned a status code in the range of 2xx or 3xx at any point in the query. To reduce the burden on the target servers a HEAD request was attempted first and if the request failed it was followed by a subsequent GET request. Of all 107,341 dark citations identified, 96,690 (90.0%) had valid URLs, 5,862 (5.5%) returned 404 errors, and 4,789 (4.5%) returned client or server errors.

## 3. Results

**Figure 1:**
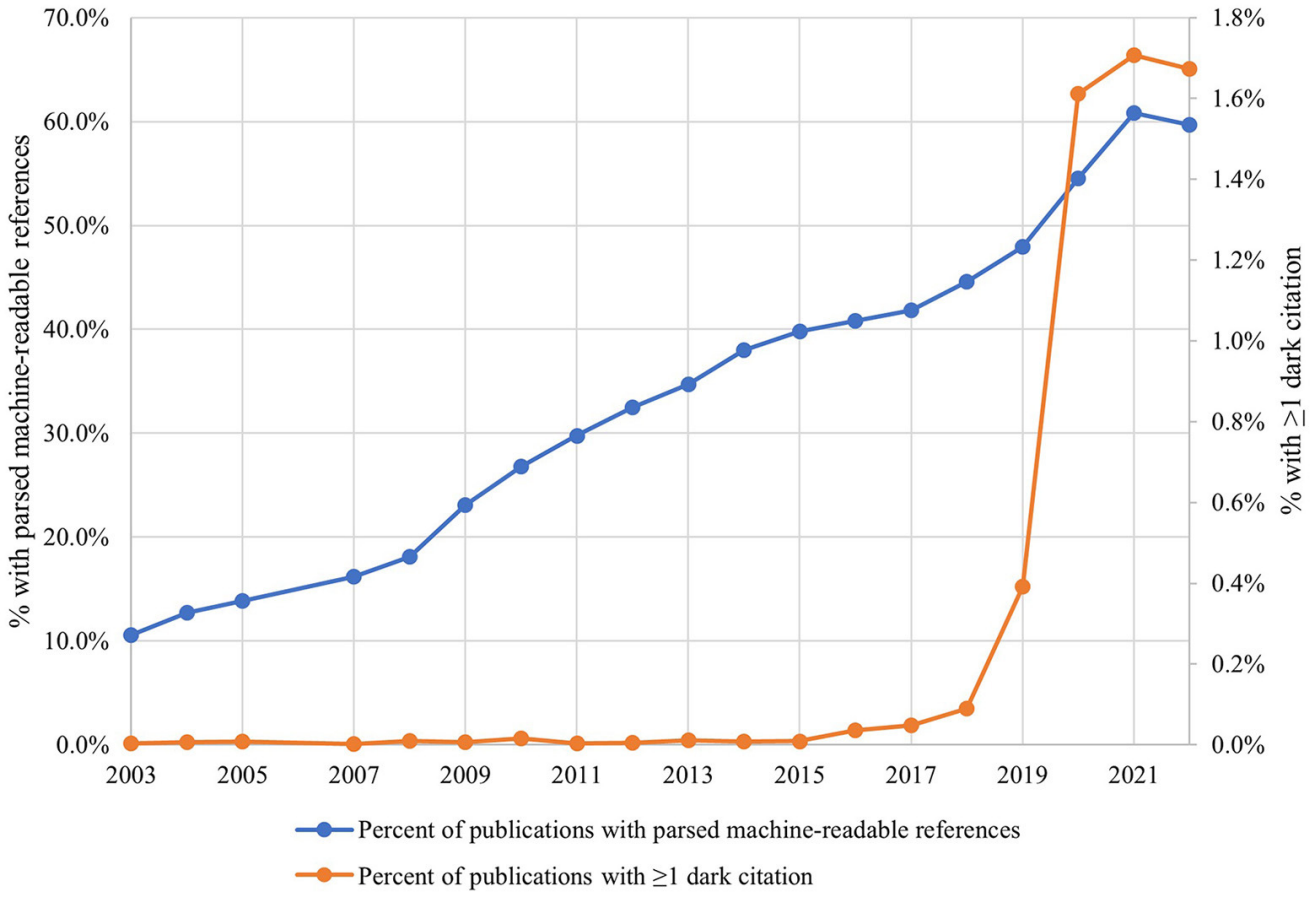
Fraction of all PubMed articles that have a parsed machine-readable reference section (blue) and fraction of all PubMed articles with a parsed machine-readable reference section that have at least one dark citation (red). The dip in reference sections of publications in the year of this analysis (2022) is likely caused by incomplete information from publishers on new publications and may resolve in the subsequent year as records are updated.

A total of 96,690 dark citations with valid URLs were identified among references cited by all publications for all years within the entire PubMed database. Figure 1 shows the percentage of all publications indexed in PubMed that include a parsed machine-readable reference section, as well as the percentage of those publications with a parsed machine-readable reference section that contain at least one dark citation, beginning in 2003. While the percentage of indexed publications with a parsed machine-readable reference section increases fairly steadily over the 20-year time period, the percentage of those that contain at least one dark citation remains consistently below 0.02% until 2016, when reaches 0.04%, and then increases dramatically beginning in 2019.

Approximately 94% of these dark citations originated from the Federal level, primarily in the Executive branch 92%; 2% were from the Legislative branch, and only 49 (<0.1%) were from the Judicial branch.^5^ Four percent were found at the state level. The remaining dark citations were found in municipal, country, tribal top-level domains. A handful of dark citations were found in true multi-level domains (e.g., the Appalachian Regional Commission, www.arc.gov) or National Labs (e.g., the Ames National Laboratory at Iowa State University, www.ameslab.gov). There were a small number of dark citations to domains operated by federally recognized tribal nations, including navajo-nsn.gov (Navajo Nation), cdatribe-nsn.gov (Coeur d'Alene Tribe), hopi-nsn.gov (Hopi Tribe), and menominee-nsn.gov (the Menominee Indian Tribe of Wisconsin). However, it is important to note that many tribes have websites within other domains, including the commercial .com domain (e.g., Eastern Band of Cherokee Indians at ebci.com or the Comanche Nation at comanchenation.com) or the non-profit.org domain (e.g., the Apache Tribe of Oklahoma at apachetribe.org). Detailed information on the breakdown of dark citations at each level of government is available in the Supplementary material.

Table 1 shows the breakdown of dark citations by federal executive agency. Among executive branch agencies, HHS accounted for the largest share (74.3%) of the total dark citations, followed by the Department of Commerce (5.5%), the Environmental Protection Agency (3.7%), the Department of Agriculture (2.7%), and the Department of Labor (2.0%). All other departments comprised <2% of the total dark citations.

Numbers and percentages of dark citations from HHS sub-agencies are displayed in Table 2. Within HHS, the CDC provided the largest share of dark citations (35.3%), followed by the Food and Drug Administration (FDA) (25.8%), the National Institutes of Health (NIH) (16%) and Center for Medicare & Medicaid Services (CMS) (6.3%). The U.S. Centers for Disease Control and Prevention (CDC) had the largest fraction of these citations (35%), followed by the Food and Drug Administration (FDA) (26%), the National Institutes of Health (NIH) (16%), the Office of the Secretary (5.7%), the Agency for Healthcare Research and Quality (AHRQ) (5.2%), the Health Resources and Services Administration (HRSA) (2.7%), and the Substance Abuse and Mental Health Services Administration (2.7%). All other HHS sub-agencies contributed <1% of all HHS dark citations.

Dark citations from CDC are displayed by Center or originating domain in Table 3. A large body of public health information came recently from COVID-19 related tables, guidelines, and reports that were not linked to a specific center (16.5%). The Centers with the largest fraction of dark citations included the NCHS (15.6%), the National Center for Chronic Disease Prevention and Health Promotion (14.8%), the National Center for HIV, Viral Hepatitis, STD, and Tuberculosis Prevention (10.8%), the National Center for Emerging and Zoonotic Infectious Diseases (8.8%), the National Center for Injury Prevention and Control (7.4%), and the National Center for Immunization and Respiratory Diseases (5.4%). All other CDC centers contributed <5% of all CDC dark citations.

Finally, from within NCHS, the divisions contributing the largest percentage of dark citations included the Division Health and Nutrition Examination Statistics (35.9% total, with 29.5% from non-report resources and 6.4% from official reports), the Division of Vital Statistics (27.1% total, with 15.9% from non-report resources and 11.2% from official reports), and the Division of Health Care Statistics (9.1% total, with 7.6% from non-report resources and 1.5% from official reports). A detailed breakdown of dark citations from NCHS is available in the Supplementary material.

## 4. Discussion

We examined the prevalence of dark citations across the biomedical literature at multiple levels of the U.S. Federal government by branch, department, agency, center, and division. As a result of focusing on biomedical literature, it was unsurprising to find the dark citations concentrated around agencies devoted to providing guidelines and public health advice to public. We focused on the entity with the largest share of dark citations at each level of federal governmental hierarchy: the federal executive branch, which comprised 92% of all U.S. governmental dark citations; HHS, which comprised 74% of dark citations from federal executive agencies; CDC, which comprised 35% of all dark citations from HHS agencies; and NCHS, which comprised 15% of dark citations from CDC centers.

A significant number of dark citations were due to publications on the SARS-CoV-2 novel coronavirus and the COVID-19 pandemic. Dark citations with COVID-related keywords (“coronavirus,” “covid,” “ncov,” “pandemic,” and “sars”) comprised 8.1% of the total, including 7.5% of federal executive, 10.2% of state, 15.5% of county, and 27.9% of municipal dark citations. The fraction of CDC citations related to COVID-19 based on these keywords was at least 19.2%, Other prominent topics based on keyword analysis included drug abuse and overdose (keywords “drug,” “opioid,” and “overdose,” 11.8% of dark citations), cancer (keyword “cancer,” 5.4% of dark citations), HIV (keywords “HIV” and “AIDS,” 3.5% of dark citations), and nutrition (keywords “diet,” “food,” “nutrition,” and “plate,” 2.0% of dark citations). Many of the most common dark citations were resources on research standards or direct references to statistics. For example, the most common dark citation (cited 345 times) was for the Study Quality Assessment Tools developed by the National Heart, Lung, and Blood Institute,^6^ while the fourth most common dark citation (cited 151 times) was the FDA's Bioanalytical Method Validation Guidance for Industry.^7^ Similarly, the second most cited resource (cited 231 times) was CDC's HIV surveillance report library^8^; citations to COVID-19 case counts^9^ (cited 132 times) and national diabetes statistics^10^ (cited 116 times) were also among the top ten. References to Census Bureau web resources comprised 3.8% of all dark citations.

The importance of reference tracking in general, and dark citations specifically, varies by the entity conducting the bibliometric analysis. Peer-reviewed journals, for example, rely on the calculated impact factor (Kaldas et al., 2020) or other citation-based metrics (Hutchins et al., 2016) as the “gold standard” by which their reach and influence on research in the field are assessed and by which they are compared to other journals. Indexed identifiers (e.g., DOIs) are essential for bibliometric analyses, as nearly all peer-reviewed publications assign one to every article they publish. Organizations such as government agencies, think tanks, advocacy groups, and other non-profit entities track references to their work as a means of demonstrating their reach, influence, and value to stakeholders, particularly donors (for non-governmental organizations) or taxpayers (as represented by legislative assemblies, for government agencies). Regardless of the specific motive, tracking usage of published materials through frequency of citation is an important means of demonstrating and quantifying impact and influence for both individuals and organizations. Thus, understanding the full reach and usage of dark citations may become more necessary as such citations become more frequent.

Using bibliometrics to track references to agency websites may have greater relevance for some agencies and less for others, as some agencies' work may not involve research publication. For example, agencies with mandates primarily related to conducting intra or extramural research such as the National Institutes of Health have a direct incentive to produce indexed products such as peer-reviewed journal articles, patents, or clinical trials (Boyack and Jordan, 2011). In contrast, agencies such as the Centers for Disease Control and Prevention or the Food and Drug Administration have additional mandates to produce science-based practice guidelines, policy documents, recommendations, or authoritative statistics. These government-produced materials were often cited directly in the reference section and are the primary focus of this analysis.

The New England Journal of Medicine's Ingelfinger rule—which stipulated that the journal would only consider a manuscript for publication if its substance has not been submitted or reported elsewhere (Angell and Kassirer, 1991) largely shaped traditional scientific norms around publishing in the latter half of the 20^th^ century (Peters, 2013). However, modern publishing and scientific consumption have challenged some of these norms, including the rise of preprints, social media, and web-only digital products. Rather than relying on traditional media to disseminate published findings, U.S. federal government agencies now work to make information easily accessible to the general public, 85% of whom own a smartphone (Pew Research Center, 2021). Additionally, information on an official agency website is considered authoritative and accepted as a reliable source of information in scientific research, and many agencies seek to make their websites the primary source and dissemination platform for their scientists' work.

Because the primary purpose of citations in a research manuscript is to demonstrate that the theoretical framework and methods on which the work is based is sound, and were drawn from authoritative sources, authors have little incentive to search for referenced information exclusively from indexed sources when dark citations such as government websites are accepted as authoritative by the scientific community. Thus, we expect these types of dark citations will only become more common. The ability to quantify and analyze dark citations will become increasingly important to the discipline of bibliometrics as scientific information dissemination norms continue to evolve.

## Data availability statement

The original contributions presented in the study are included in the article/Supplementary material, further inquiries can be directed to the corresponding author.

## Author contributions

TH downloaded all data from PubMed, extracted and deduplicated reference information, and compiled all dark citations used for the analysis. JK categorized all dark citations and produced summary statistics for the analysis. JK, JA-D, and TH drafted the manuscript. JK and JA-D created the tables. All authors contributed to the article and approved the submitted version.

## Footnotes

^1^Pendlebury DA. *Using Bibliometrics in Evaluating Research* (White Paper). Philadelphia, PA, Department R.

^2^ https://ftp.ncbi.nlm.nih.gov/

^3^A listing of agencies within the U.S. Department of Health and Human Services, which is a cabinet-level federal executive agency, can be found at https://www.usa.gov/federal-agencies/u-s-department-of-health-and-human-services.

^4^ https://home.dotgov.gov/data/

^5^An overview of how the U.S. federal government is organized is available at https://www.usa.gov/branches-of-government.

^6^ https://www.nhlbi.nih.gov/health-topics/study-quality-assessment-tools

^7^ https://www.fda.gov/files/drugs/published/Bioanalytical-Method-Validation-Guidance-for-Industry.pdf

^8^ http://www.cdc.gov/hiv/library/reports/hiv-surveillance.html

^9^ https://www.cdc.gov/coronavirus/2019-ncov/cases-updates/cases-in-us.html

^10^ https://www.cdc.gov/diabetes/pdfs/data/statistics/national-diabetes-statistics-report.pd
