# Supplementary material for: Dark citations to Federal resources and their contribution to the public health literature
Source: Front Res Metr Anal. 2023 Aug 29;8:1235208. doi: 10.3389/frma.2023.1235208 (PMC10497748; doi:10.3389/frma.2023.1235208)
Supplement: Supplementary file 1 [file Table_1.DOCX]

# Supplemental Tables

| **Citation Type** | **Dark Citation Text** | **Title of Citing Paper** | **PMID** | **Journal** |
| --- | --- | --- | --- | --- |
| Guideline | Centers for Disease Control and Prevention. (2017). CDC guidelines for prescribing opiates for chronic pain: Fact sheet. Retrieved from https://www.cdc.gov/drugoverdose/pdf/guidelines_at-a-glance-a.pdf. | Treating chronic pain and opioid misuse disorder among underserved populations in Colorado | [31169790](https://pubmed.ncbi.nlm.nih.gov/31169790/) | J Am Assoc Nurse Pract |
| Fact Sheet | Centers for Disease Control and Prevention. Pelvic inflammatory disease (PID) – CDC fact sheet. Available at: https://www.cdc.gov/std/pid/stdfact-pid-detailed.htm. Retrieved February 15, 2019. | Laparoscopic Compared With Open Surgery for Severe Pelvic Inflammatory Disease and Tubo-Ovarian Abscess | [31135738](https://pubmed.ncbi.nlm.nih.gov/31135738/) | Obstet Gynecol |
| Manual | National Health and Nutrition Examination Survey (NHANES) anthropometry procedures manual, January 2009. Available at: http://www.cdc.gov/nchs/data/nhanes/nhanes_09_10/BodyMeasures_09.pdf. Retrieved December 21, 2015. | Pregnancies After the Diagnosis of Mild Gestational Diabetes Mellitus and Risk of Cardiometabolic Disorders | [28079773](https://pubmed.ncbi.nlm.nih.gov/28079773/) | Obstet Gynecol |
| Web Page | Centers for Disease Control and Prevention. Adult BMI. http://www.cdc.gov/healthyweight/assessing/bmi/adult_bmi/. Published May 15, 2015. Accessed June 15, 2015. | Gestational Weight Gain and Breastfeeding Outcomes in Group Prenatal Care | [27428789](https://pubmed.ncbi.nlm.nih.gov/27428789/) | J Midwifery Womens Health |

Supplemental Table 1: Examples of dark citations, including the source publication, journal, and citation text as it appears in the reference section. The table highlights four different types of products commonly found in dark citations: guidelines, fact sheets, manuals, and web pages.

| **U.S. Government Level** | **Dark citations** | **Percent** |
| --- | --- | --- |
| Federal, Executive | 96,167 | 92.1% |
| State | 4,604 | 4.4% |
| Federal, Legislative | 1,980 | 1.9% |
| Other: National Labs | 661 | 0.6% |
| Multi-level | 72 | 0.1% |
| Municipal | 662 | 0.6% |
| County | 181 | 0.2% |
| Federal, Judicial | 49 | <0.1% |
| Tribal | 9 | <0.1% |

Supplemental Table 2: Number and percent of dark citations from PubMed identified across the US Federal, State, local, and tribal governments that include a .gov URL.

| **Division** | **Acronym** | **Dark citations** | **Percent** |
| --- | --- | --- | --- |
| **Web pages and non-report resources** | | | |
| Division of Health and Nutrition Examination Statistics | DHANES | 1,150 | 29.49% |
| Division of Vital Statistics | DVS | 621 | 15.92% |
| Division of Health Care Statistics | DHCS | 296 | 7.59% |
| FastStats (cross-divisional) |  | 262 | 6.72% |
| Division of Health Interview Statistics | DHIS | 246 | 6.31% |
| Division of Analysis and Epidemiology | DAE | 204 | 5.23% |
| Research Data Center | RDC | 12 | 0.31% |
| Office of the Director | OD | 11 | 0.28% |
| Office of Information Technology | OIT | 5 | 0.13% |
| Division of Research Methodology | DRM | 3 | 0.08% |
| **Reports: NHSRs, NVSRs, SRs, data briefs** | | | |
| Division of Vital Statistics | DVS | 436 | 11.18% |
| Division of Health and Nutrition Examination Statistics | DHANES | 248 | 6.36% |
| Division of Analysis and Epidemiology | DAE | 92 | 2.36% |
| Division of Health Care Statistics | DHCS | 60 | 1.54% |
| Division of Health Interview Statistics | DHIS | 59 | 1.51% |
| Division of Research Methodology | DRM | 1 | 0.03% |
| Health E-stats |  | 79 | 2.03% |
| Press releases |  | 58 | 1.49% |
| Other/Unknown |  | 57 | 1.46% |

Supplemental Table 3: Number and percent of dark citations from PubMed within National Center for Health Statistics (NCHS), by division or office split into reports and non-reports like webpages and other resources.
